# Natural Anæsthesia

**Published:** 1858-03

**Authors:** 


NATURAL ANÆSTHESIA.—"Starting and looking half round, I saw the lion just in the act of springing upon me. I was upon a little height: he caught my shoulder as he sprang, and we came to the ground together. Growling horribly close to my ear, he shook me as a terrier does a rat. The shock produced a stupor similar to that which seems to be felt by a mouse after the first shake of the cat. It caused a sort of dreaminess, in which there was no sensation of pain nor feeling of terror, though quite conscious of all that was happening. It was like what patients partially under the influence of chloroform describe, who see all the operation but feel not the knife. This singular condition was not the result of any mental process. The shake annihilated fear, and allowed no sense of horror in looking around at the beast. This peculiar state is probably produced in all animals killed by the carnivora; and, if so, is a merciful provision by our benevolent Creator for lessening the pain of death."—*Livingstone's Travels.*

